# Mediating pathways in the socio-economic gradient of child development: Evidence from children 6–42 months in Bogota

**DOI:** 10.1177/0165025415626515

**Published:** 2016-06-17

**Authors:** Marta Rubio-Codina, Orazio Attanasio, Sally Grantham-McGregor

**Affiliations:** 1 Institute for Fiscal Studies, London, UK; 2 Inter-American Development Bank, Washington DC, USA; 3 University College London, London, UK; 4 Institute of Child Health, London, UK

**Keywords:** Socio-economic gap, early childhood development, parental education, home environment

## Abstract

Research has previously shown a gap of near 0.5 of a standard deviation (SD) in cognition and language development between the top and bottom household wealth quartile in children aged 6–42 months in a large representative sample of low- and middle-income families in Bogota, using the Bayley Scales of Infant and Toddler Development. The gaps in fine motor and socio-emotional development were about half that size. Developmental deficits increased with age. The current study explored the associations amongst child development, household socio-economic status (SES), and a set of potential mediating variables—parental characteristics, child biomedical factors, and the quality of the home environment—in this sample. We ran mediation tests to quantify the contribution of these variables to the SES gap, and explored the role of age as a moderator. Parental education, particularly maternal education, and the quality of the home environment mediated the SES gap in all outcomes examined. Height-for-age mediated a small amount of the deficit in language scales only. More educated mothers provided better home stimulation than less educated mothers and the home environment partly mediated the effect of maternal education. These results suggested that in interventions aimed at promoting child development, those focusing on the quality of the home environment should be effective.

The negative effects of growing up in disadvantaged environments on child development are well documented. Cross-sectional studies in low- and middle-income countries showed cognitive and language deficits associated with poverty for children 36 months and older. These deficits increased with age. See papers by Schady et al. (2015) for five Latin American countries; Fernald, Weber, Galasso, and Ratsifandrihamanana (2011) for Madagascar; and Naudeau, Martinez, Premand, and Filmer (2011) for Cambodia and Mozambique. For children younger than 24 months, Fernald, Kariger, Hidrobo, and Gertler (2012) showed that wealth and maternal education were related to mothers’ reports on communication, gross motor and personal-social development in rural India, Indonesia, Peru, and Senegal.

Rubio-Codina, Attanasio, Meghir, Varela, and Grantham-McGregor (2015) have recently investigated the effect of socio-economic status (SES) on developmental outcomes for a sample of children 6–42 months in low- and middle-income families in Bogota, Colombia. Child development was assessed using the *Bayley Scales of Infant and Toddler Development*, third edition (Bayley-III) (Bayley, 2006). The authors demonstrated a SES gap of near 0.5 of a standard deviation (SD) in cognition and language between children in the top and bottom quartile of the within-sample household wealth distribution. Gaps in fine motor and socio-emotional development were about half that size, whereas that in gross motor was not statistically significant. Gaps increased substantially with age for cognition and receptive language.

A growing body of evidence thus indicates that children from diverse socio-economic backgrounds experience markedly different developmental trajectories. These deficits in the early years are known to be associated with lower cognition and school attainment, and subsequent lower wages and well-being later on in life; hence contributing to the perpetuation of poverty (Naudeau et al., 2011; Walker et al., 2011). Nonetheless, few studies have *simultaneously* explored the role of other determinants of child development alongside wealth, such as parental characteristics or the home environment. Understanding their contribution to the wealth gap is important in order to identify policies that can reduce developmental deficits. A recent exception is the study by Hamadani et al. (2014), who followed a panel of poor children in rural Bangladesh from birth to 5 years of age. The authors documented that the increasing wealth gap was mediated by parental education, birth weight and growth in the first 24 months, and by home stimulation through 5 years of age.

Following the approach in Hamadani et al. (2014), we used the data from the Bogota study (described above) to examine the relationships amongst child development, household SES, and a set of potential mediating variables including parental characteristics, firstborn child, child height-for-age, gestational age, birth weight, and the quality of the home environment. These variables have been shown to be associated with child development and household SES earlier (Hamadani et al., 2014; Walker et al., 2011). We explored whether these associations were significant in the Bogota data and the extent to which these variables mediated the relationships between household SES and developmental outcomes documented in Rubio-Codina et al. (2015). We investigated mediation separately for all Bayley-III scales which exhibited statistically significant SES gradients to explore specificity of effects by developmental domain. Recent studies in developmental neuroscience showed that different regions in the brain were affected by SES differently (Hackman, Farah & Meaney, 2010). Finally, we explored the role of age as a moderator in the mediation analysis. The age effect may not be linear. For example, growth in the first 2 years was more highly related to developmental deficits than later growth in various cohort studies (Hamadani et al., 2014; Martorell et al., 2010). The mediation analysis we performed could inform the design of well-targeted, timely interventions that promote early childhood development.

## Method

### Participants

The study enrolled an age- and sector-stratified random sample of 1,533 children 6–42 months between March and August 2011, whose parents consented to participate. These children lived in 497 blocks in the lowest three socio-economic strata of Bogota. Each neighborhood is classified into one of six socio-economic sectors by the city government, depending on location and quality of infrastructure and housing, as a mechanism to target public services’ subsidies. The first three sectors account for 8.1%, 37.3%, and 38.2%, respectively, of Bogota’s population. Neighborhoods in the bottom two are considered poor, while those in the third sector are considered lower-middle class. Yet, there is substantial heterogeneity in household SES within sector (Rubio-Codina et al., 2015).

Within sector, blocks were randomly selected weighting by the proportion of women in fertile age. While the original design included sector four households, we dropped it after 2 months in the field due to the high participation refusal rate (but kept the 12 children already tested to maximize the variance in the household wealth distribution). We did not assess children below 6 months given the low predictive ability of measures at these ages. We excluded one child with mental disabilities, a pair of twins, and four children (at random) in households with more than one available child. Rubio-Codina et al. (2015) provided further details on the study design.

### Procedures and Measurements

Child’s gestational age, birth weight and health history were collected by caregiver report during a household survey administered by an interviewer. The survey also included basic household socio-economic information (demographic composition, education and employment of members, dwelling characteristics, assets, etc.) and measures of quality of the home environment using UNICEF’s *Family Care Indicators* (FCI) (Frongillo, Sywulka & Kariger, 2013). Specifically, we recorded, by observation, the number of books for adults, newspapers/magazines, and the toys the child usually played with by type; and by report, the play activities the child and an adult engaged in over the week prior to the survey. Reliability data was not available for these variables.

On average, 5–6 days after the household survey, children were assessed on the Bayley-III. Testing took place in the presence of the caregiver in the nearest local library or child care center. The cognitive, language (receptive and expressive) and motor (fine and gross) scales were directly administered. The socio-emotional scale was collected by caregiver reports (89% mothers) on 35 5-point rating items. The adaptive behavior questionnaire was not administered because of time constraints. Also, some items were culturally inappropriate (e.g. “turns television on and off” or “walks on sidewalk rather than street”). The test was translated to Spanish and back-translated to English. Minor modifications were made to the translated version after piloting to ensure linguistic and functional equivalence. Test–retest reliability (intra-class correlation, *r*) after 2–11 days (median = 8 days) was good (*r* = [0.96–0.98] for the cognitive, language, and motor scales; *r* = 0.88 for the socio-emotional questionnaire, *n* = 20). Internal consistency was also good: Chronbach’s α = 0.86 for the socio-emotional scale and α = [0.96–0.97] for the cognitive, language, and motor scales. Testers held undergraduate degrees in Psychology and undertook 6 weeks’ training, including practice sessions in couples with children similar to study participants. Practice testing continued until inter-rater reliabilities *r* > 0.9 were obtained on each scale, between each pair of testers, and between tester and trainer. Furthermore, 5% of the measurements were supervised by the trainer (*r* > 0.9 in 84% of the cases, mean *r* = 0.95) and corrective feedback was given when appropriate. The trainer had a master degree in Psychology and prior experience in training.

The child’s height and weight were measured after the Bayley-III, following WHO protocols (WHO, 1983).

### Statistical Analysis

Following Rubio-Codina et al. (2015), we standardized developmental scores internally using the empirical age-conditional mean and SD estimated using regression methods. Polynomials in age of varying order, depending on the scale, were fitted. This procedure is less sensitive to outliers and small sample sizes within age category than using months-of-age specific means and SDs (as commonly done to standardize developmental scores from developing countries). We also re-constructed the household wealth index as the standardized first principal component of dwelling characteristics (e.g. high-quality floors, garage, shared kitchen) and assets (e.g. fridge, car, computer), with eigenvalue of 8.62 and accounting for 43.09% of the variance. Polychoric correlations were used to allow comparability of loadings given that the index combined continuous and categorical variables.

Similarly, we used polychoric principal component analysis on the items of the FCI to construct a summary measure of the level of stimulation in the home (quality of the home environment index). We included the number of books for adults, the number of newspapers/magazines, and whether the household had a particular toy or provided a particular form of stimulation (listed in Panel III in Table 1). We excluded “taking child outside home place or going for a walk” because of the low loading, possibly due to the little variability in the variable (89% of caregivers reported “yes”). The resulting first principal component had an eigenvalue of 6.08 and explained 37.99% of the variance.

**Table 1.** Sample Characteristics.

		**Mean**	**SD**
I. Child characteristics			
	6–18 months, (*n* = 451)	33.91%	
	19–30 months, (*n* = 460)	34.59%	
	31–42 months, (*n* = 419)	31.50%	
	Girls, (*n* = 655)	49.25%	
	Gestational age, weeks	38.38	2.19
	Birth weight, g	3036.33	0.51
	*Z*-score height-for-age	−1.10	1.08
	Firstborn	50.38%	
II. Parental and household characteristics			
	Education years mother	10.28	3.37
	Education years father	8.39	3.94
	Age mother	26.94	6.65
	Mother paid employment	35.71%	
	Teenage pregnancy (mother)	13.31%	
	Household size	4.66	1.60
	Grandmother lives in household	26.84%	
III. Quality of home environment index, components			
	Books for adults (none, 1 or 2, 3–14, more than 15)	1.36	1.19
	Newspapers and magazines (none, 1 or 2, 3–10, more than 11)	1.29	1.27
	Toys that make or play music	69.32%	
	Toys or objects meant for stacking, constructing or building	48.87%	
	Things for drawing, writing, coloring, and painting	64.74%	
	Toys for moving around	93.08%	
	Toys to play pretend games	56.47%	
	Picture books for children	30.15%	
	Drawing books for children	43.08%	
	Toys for learning shapes and colors	42.71%	
	Reading books or looking at picture books	42.56%	
	Telling stories to child	30.60%	
	Singing songs to/with child	87.14%	
	Playing with child with her toys	74.51%	
	Spending time with child scribbling, drawing, or coloring	50.83%	
	Spending time with child naming things or counting	63.38%	

*Notes.* (*N* = 1,330). Missing data for child’s height-for-age (*N* = 1,327), mother’s education (*N* = 1,324), and father’s education (*N* = 1,232). Data are means unless stated otherwise (% or percentages).

Missing levels of mother’s and father’s education (0.45% and 7.37%) and child’s height-for-age (0.23%) were replaced with the median value of the variable in the corresponding quartile of the wealth distribution. This replacement was accounted for in the analysis.

**Figure 1.**
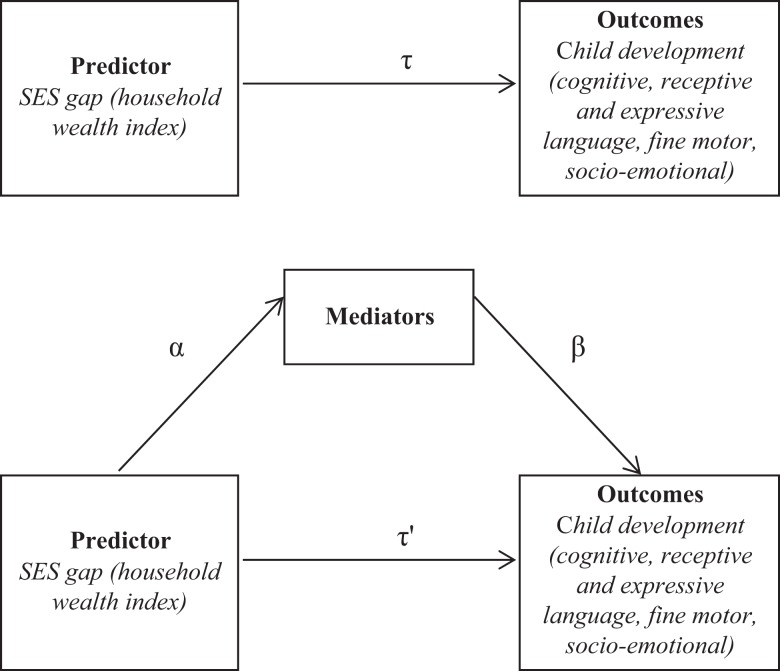
Mediation Model. Notes: Control variables are child's age, sex, and tester dummies

Figure 1 depicts a standard path diagram for mediation (MacKinnon, Lockwood, Hoffman, West & Sheets, 2002) adapted to our model. A mediator has to be related to both the predictor and the outcomes. We therefore examined the correlations among the potential mediators considered, household SES, and child development outcomes. Gestational age and birth weight were not significantly associated with the predictor (household SES) and were discarded. Maternal age and teenage pregnancy were also excluded because they were not significantly associated with the outcomes.

We then examined whether the remaining variables—i.e. parental education, firstborn child, height-for-age, and the quality of the home environment—were potential mediators.

We started by replicating the analysis in Rubio-Codina et al. (2015) for each developmental domain where the SES gap between children 6–42 months in the top and bottom quartile was statistically significant (i.e. all except gross motor). We examined differences in developmental scores by wealth quartiles controlling for child’s age and sex, and tester effects, using multiple regression analysis and clustering standard errors at the residential block level (i.e. allowing for correlation within the residential blocks, the primary sampling unit) for all subjects available. These regressions offered the estimated association of the predictor (SES gap) on outcomes (developmental scores) before controlling for any potential mediators, *τ* in Figure 1. We then estimated the *direct association* between the SES gap and developmental outcomes controlling for the proposed mediators, which were entered in four sequential steps. Firstly, we entered mother’s and father’s years of education (standardized within the sample). Secondly, we entered a dummy for firstborn child. Thirdly, we entered height-for-age *Z*-score (computed using the WHO Anthro software, 2011) as a measure of the child’s nutritional status. In a fourth and final step, we entered the quality of the home environment index. Each step offered an estimate of *τ’* in Figure 1. Introducing potential mediators sequentially and in this order assumed that parental education occurred before the child’s birth, and affected fertility decisions, the child’s height-for-age, and the level of stimulation in the home. Alternative specifications that considered different ordering of entry of the potential mediators, or included each set of potential mediators separately yielded similar results (not reported).

For each step, we formally tested whether these mediating factors significantly reduced the association between the SES gap on Bayley-III scores—i.e. *Ho: τ− τ’ = 0* (no mediation). In an OLS regression model, testing the statistical significance of (*τ − τ’*) is algebraically equivalent to testing that of *αβ* (MacKinnon, Warsi & Dwyer, 1995), known as the *indirect association* between the predictor and outcome via the mediator (Dearing & Hamilton 2006). These tests were constructed by bootstrap methods (Efron, 1982) to account for the fact that different regressions were estimated on the same sample (500 replications). As in Rubio-Codina et al. (2015), child’s age and sex, and tester effects were controlled for in all regressions, and standard errors (hence, confidence intervals and *p*-values) were clustered at the residential block level.

Using similar analysis, we further examined whether the effects of mother’s education on child development were mediated through height-for-age and home stimulation. Both height-for-age and the home environment were correlated with maternal education.

We first examined mediation following this approach for all children with Bayley-III data. We then examined whether the effect of the mediators of the SES effect on child development varied by age by replicating the analysis for three 12-month-of-age groups. We used bootstrap methods to test whether the mediating effects (*τ− τ’*) were statistically different within each age subgroup.

## Results

### Sample Characteristics

Table 1 shows the mean and SD for a selection of child, parental and household characteristics for the 1,330 children (86.8%) who came for Bayley-III testing. Lack of Bayley-III assessment was associated with younger mothers, more young siblings, and childcare attendance, suggesting caregivers could not afford the time to take children to the center for the test. Nonetheless, the SES distributions of children in the original and Bayley-III samples were comparable: a chi- square test of goodness of fit cannot reject the null that the proportion of children not tested was equally distributed across wealth quartiles (*p* = 0.341).

As shown in Panel I of Table 1, the children tested were well distributed across age groups and gender. The average gestational age and birth weight were in the normal range, and the prevalence of stunting (-2SD below median WHO height-for-age *Z*-score) was 17.59%. On average, mothers were more educated than fathers (Panel II). While 11.62% of mothers had primary education completed or less (vs. 20.13% of fathers), fathers were more likely to complete secondary education conditional on having started. Panel III in Table 1 shows variation in the components of the quality of the home environment index.

Table 2 reports the correlation coefficients amongst the predictor, potential mediators, and outcomes. All variables correlated with SES and with at least three of the developmental domains. Maternal education was also correlated with height-for-age (*r =* 0.059, *p* < 0.05) and the home environment index (*r =* 0.423, *p* < 0.001). Hence, all criteria for mediation analysis were met. Paternal education was also correlated with the home environment index (*r* = 0.235, *p* < 0.001), although the correlation was smaller than with maternal education. As expected, maternal and paternal education were correlated (*r* = 0.346, *p* < 0.001).

**Table 2.** Correlations Across Predictor, Developmental Outcomes, and Potential Mediators.

	Cognition	Receptive language	Expressive language	Fine motor	Socio-emotional	SES index
Predictor						
SES index	0.225***	0.195***	0.218***	0.119***	0.115***	1
Potential mediators						
Mother’s education	0.223***	0.241***	0.230***	0.136***	0.079**	0.473***
Father’s education	0.141***	0.110***	0.138***	0.046	0.110**	0.317***
Firstborn	0.061*	0.102***	0.111***	−0.004	0.028	0.117***
Height-for-age	0.051*	0.076**	0.076**	0.061*	−0.016	0.096***
Home environment index	0.245***	0.255***	0.229***	0.137***	0.181***	0.423***

*Notes.* (*N* = 1,330). All variables are *Z*-scores, except for firstborn (indicator). ***p < 0.001, **p < 0.01, *p < 0.05

### Mediators of the SES Gap

Table 3 reports the coefficient estimates and clustered 95% confidence intervals (CIs) of the multiple regression analysis on Bayley-III *Z*-scores by steps for all children in the sample. For each panel, which refers to a different developmental domain, Step 0 shows the size of the SES gap (*τ*) in developmental scores (reported in Rubio-Codina et al., 2015). Each subsequent step shows the size of the SES gap after controlling for a new set of potential mediators and the coefficients on these variables (*τ’* and β in Figure 1).

**Table 3.** Regression Coefficients and 95% CIs for the Effect of the SES Gap and Potential Mediators on Bayley-III *Z*-Scores. All Children 6–42 Months (*N* = 1,330).

		Step 0		Step 1		Step 2		Step 3		Step 4	
		τ	(95% CI)	τ’, β	(95% CI)	τ’, β	(95% CI)	τ’, β	(95% CI)	τ’, β	(95% CI)
I. Cognition											
Step 0:	SES gap	0.53	(0.38–0.69)***	0.33	(0.16–0.50)***	0.32	(0.15–0.50)***	0.31	(0.14–0.49)***	0.21	(0.04–0.39)*
Step 1:	Mother’s education			0.12	(0.05–0.20)***	0.12	(0.04–0.19)**	0.12	(0.04–0.19)**	0.07	(−0.00–0.14)
	Father’s education			0.07	(0.01–0.12)*	0.07	(0.01–0.13)*	0.07	(0.01–0.13)*	0.06	(−0.00–0.11)
Step 2:	Firstborn					0.06	(−0.04–0.16)	0.05	(−0.05–0.15)	0.04	(−0.07–0.14)
Step 3:	Height-for-age							0.04	(−0.01–0.09)	0.04	(−0.01–0.09)
Step 4:	Home environment index									0.16	(0.10–0.22)***
II. Receptive language											
Step 0:	SES gap	0.42	(0.29–0.54)***	0.17	(0.03–0.31)*	0.16	(0.02–0.30)*	0.15	(0.01–0.28)*	0.05	(−0.09–0.18)
Step 1:	Mother’s education			0.18	(0.11–0.25)***	0.16	(0.10–0.23)***	0.16	(0.10–0.23)***	0.12	(0.05–0.19)**
	Father’s education			0.03	(−0.03–0.09)	0.04	(−0.02–0.10)	0.04	(−0.02–0.10)	0.03	(−0.03–0.08)
Step 2:	Firstborn					0.13	(0.02–0.23)*	0.12	(0.01–0.22)*	0.10	(−0.01–0.20)
Step 3:	Height-for-age							0.07	(0.02–0.11)**	0.06	(0.02–0.11)**
Step 4:	Home environment index									0.16	(0.10–0.22)***
III. Expressive language											
Step 0:	SES gap	0.49	(0.35–0.64)***	0.26	(0.10–0.42)**	0.25	(0.09–0.41)**	0.24	(0.07–0.40)**	0.15	(−0.01–0.31)
Step 1:	Mother’s education			0.16	(0.09–0.22)***	0.14	(0.07–0.21)***	0.14	(0.07–0.21)***	0.10	(0.03–0.17)**
	Father’s education			0.06	(−0.00–0.11)	0.07	(0.01–0.12)*	0.06	(0.01–0.12)*	0.05	(−0.01–0.11)
Step 2:	Firstborn					0.15	(0.05–0.26)**	0.14	(0.04–0.25)**	0.13	(0.02–0.23)*
Step 3:	Height-for-age							0.05	(0.01–0.09)**	0.05	(0.01–0.09)**
Step 4:	Home environment index									0.13	(0.07–0.20)***
IV. Fine motor											
Step 0:	SES gap	0.26	(0.11–0.42)**	0.14	(−0.04–0.32)	0.14	(−0.03–0.32)	0.13	(−0.05–0.31)	0.06	(−0.12–0.23)
Step 1:	Mother’s education			0.10	(0.03–0.17)**	0.10	(0.03–0.17)**	0.10	(0.03–0.17)**	0.07	(−0.00–0.14)
	Father’s education			0.00	(−0.05–0.06)	−0.00	(−0.06–0.05)	−0.00	(−0.05–0.05)	−0.01	(−0.06–0.04)
Step 2:	Firstborn					−0.04	(−0.15–0.06)	−0.05	(−0.16–0.05)	−0.07	(−0.17–0.03)
Step 3:	Height-for-age							0.06	(0.01–0.10)*	0.06	(0.01–0.10)*
Step 4:	Home environment index									0.11	(0.05–0.18)**
V. Socio-emotional											
Step 0:	SES gap	0.27	(0.12–0.42)***	0.19	(0.04–0.34)*	0.18	(0.03–0.33)*	0.19	(0.04–0.34)*	0.12	(−0.03–0.27)
Step 1:	Mother’s education			0.02	(−0.04–0.08)	0.02	(−0.04–0.08)	0.02	(−0.04–0.08)	−0.01	(−0.08–0.05)
	Father’s education			0.07	(0.02–0.13)**	0.08	(0.02–0.13)**	0.08	(0.02–0.13)**	0.07	(0.01–0.12)**
Step 2:	Firstborn					0.03	(−0.08–0.15)	0.04	(−0.08–0.16)	0.03	(−0.09–0.14)
Step 3:	Height-for-age							−0.03	(−0.07–0.02)	−0.03	(−0.07–0.02)
Step 4:	Home environment index									0.11	(0.06–0.17)***

*Notes.* ***p < 0.001, **p < 0.01, *p < 0.05. For each outcome (panel), columns represent separate regressions. All variables are *Z*-scores, except for firstborn (indicator). All regressions control for wealth quartile dummies, child’s age and sex, and tester effects. 95% Confidence Intervals (CIs) are adjusted for clustering at the block level.

In Step 1, the mother’s education showed a positive significant association with child development for all domains except socio-emotional development. The father’s education, however, was significantly associated with cognitive and socio-emotional development. In Step 2, being the firstborn child had a positive significant association with receptive and expressive language only. In Step 3, height-for-age showed positive significant associations with receptive and expressive language and with fine motor development. In Step 4, the quality of the home environment index was significantly associated with all developmental domains. By the final step, the initial developmental gaps associated to SES were substantially reduced. The SES gap in cognition decreased by 60%, but remained significant in the fourth step. In both language scales and in socio-emotional development the SES gap was no longer significant by Step 4, whereas for fine motor, it was no longer significant after including parental education.

Panel I in Table 4 reports results of the test for mediation, (*τ− τ’*), for each set of potential mediators. The test for mediation of parental education was significant for all domains^1^. Entering parental education in Step 1 reduced the effects of SES by 58% for receptive language, 48% for expressive language and fine motor, 39% for cognition, and 32% for socio-emotional development. In Step 2, the reductions in the SES effect on development after introducing firstborn were not statistically significant. Therefore, firstborn was not a mediator. Entering the child’s height-for-age (Step 3) significantly reduced the size of the SES gap, controlling for parental education and firstborn, by 6% and 11% in expressive and receptive language, respectively. The inclusion of the quality of the home environment index in Step 4 significantly reduced the effect of the SES gap on all domains, conditional on the other potential mediators. Specifically, it reduced the proportion of the remaining variance in the SES gap by 67% for receptive language, 38% for socio-emotional development, 35% for expressive language, and 33% for cognition. The effect of the home environment was also significant for fine motor development, although the SES gap was already not significant after including parental education in Step 1.

**Table 4.** Change in Effects on Bayley-III *Z*-Scores after the Inclusion of Potential Mediators. All Children 6–42 Months (*N*=1,330).

	Step 1: Mother and father education entered		Step 2: Firstborn entered		Step 3: Height-for-age entered		Step 4: Home environment index entered	
	(τ−τ_1_’)	(95% CI)	(τ_1_’−τ_2_’)	(95% CI)	(τ_2_’−τ_3_’)	(95% CI)	(τ_3_’−τ_4_’)	(95% CI)
I. Change in SES gap								
Cognition	0.205	(0.119–0.311)***	0.004	(−0.003–0.017)	0.010	(−0.003–0.027)	0.101	(0.063–0.142)***
Receptive language	0.243	(0.151–0.349)***	0.008	(−0.002–0.023)	0.018	(0.004–0.036)*	0.101	(0.061–0.150)***
Expressive language	0.234	(0.146–0.347)***	0.010	(−0.003–0.029)	0.014	(0.002–0.028)*	0.085	(0.040–0.133)***
Fine motor	0.122	(0.037–0.233)**	−0.003	(−0.014–0.005)	0.015	(0.002–0.033)	0.072	(0.030–0.113)***
Socio-emotional	0.085	(0.004–0.166)*	0.002	(−0.005–0.016)	−0.007	(−0.022–0.005)	0.072	(0.034–0.114)***
II. Change in mother’s education effect								
Cognition			–	–	–	–	0.046	(0.029–0.065)***
Receptive language			0.016	(0.003–0.031)*	0.001	(−0.003–0.006)	0.046	(0.030–0.063)***
Expressive language			0.019	(0.006–0.033)*	0.000	(−0.003–0.005)	0.038	(0.019–0.058)***
Fine motor			–	–	0.000	(−0.003–0.005)	0.032	(0.014–0.049)***

*Notes.* ***p<0.001, **p<0.01, *p<0.05. Bootstrapped 95% Confidence Intervals (CIs) adjusted for clustering at the block level (*n*=500 replications).

Observation of the changing size of the coefficients on maternal education across steps showed that these were reduced in cognition, both languages and fine motor, particularly when the home environment entered (Step 4). We therefore examined whether the other potential mediators mediated the effect of maternal education whenever they significantly entered the regressions in Steps 2–4. Panel II in Table 4 shows that the home environment significantly reduced the association between maternal education and cognition, receptive and expressive language, and fine motor development, from 25% to 42%, depending on the scale. Firstborn mediated the effect of maternal development on both language scales. However, height-for-age was not a mediator of the effect of maternal education on development.

### Age as a Moderator

Supplementary Table 1 in the web Appendix shows the correlations amongst predictor, potential mediators, and outcomes by age groups. As expected, both the size and significance level of these correlations increased with age. Step-wise analyses by age groups are reported in Supplementary Tables 2–4. Text Table 5 reports the coefficients and CIs for the change in the size of the SES effect on development after the inclusion of the potential mediators by age subgroups (in Panels). As before, Step 0 shows the size of the SES gap (*τ*). Steps 2 and 3 are not reported because none of the mediation effects of firstborn or height-for-age by age group were statistically significant. The size of the SES gap increased with age and mediation occurred primarily amongst children in the middle and older age groups. In children 6–18 months, the SES gap was generally small and not significant for cognition and receptive language. For receptive language, there was a significant effect from parental education and the home environment; however, the SES gap was already not significant in Step 0. Introducing parental education reduced the effect of SES on expressive language, but the potential mediators were not significant (Supplementary Table 2).

**Table 5.** Change in SES Gap on Bayley-III *Z*-Scores after the Inclusion of Potential Mediators, (τ-τ’), by Age Group.

	Step 0: SES gap		Step 1: Mother and father education entered		Step 4: Home environment index entered	
	τ	(95% CI)	(τ−τ_1_’)	(95% CI)	(τ_3_’−τ_4_’)	(95% CI)
I. Children 6–18 months (*N* = 451)						
Cognition	0.26	(−0.01–0.53)	0.013	(−0.122–0.155)	0.048	(−0.008–0.120)
Receptive language	0.20	(−0.04–0.45)	0.176	(0.063–0.293)**	0.059	(0.012–0.134)*
Expressive language	0.43	(0.16–0.70)**	0.149	(0.005–0.298)*	0.012	(−0.042–0.079)
Fine motor	0.25	(0.01–0.49)*	0.001	(−0.157–0.171)	0.039	(−0.008–0.117)
Socio-emotional	0.30	(0.03–0.57)*	0.055	(−0.080–0.189)	−0.005	(−0.056–0.047)
II. Children 19–30 months (*N* = 460)						
Cognition	0.55	(0.32–0.78)***	0.251	(0.063–0.447)**	0.147	(0.072–0.239)***
Receptive language	0.30	(0.06–0.55)*	0.195	(0.025–0.377)*	0.172	(0.087–0.274)***
Expressive language	0.41	(0.17–0.65)***	0.199	(0.048–0.373)*	0.149	(0.066–0.257)***
Fine motor	0.13	(−0.09–0.36)	0.056	(−0.132–0.238)	0.094	(−0.000–0.182)**
Socio-emotional	0.20	(−0.04–0.45)	0.066	(−0.102,0.229)	0.165	(0.079–0.267)***
III. Children 31–42 months (*N* = 419)						
Cognition	0.81	(0.55 –1.08)***	0.367	(0.204–0.535)***	0.100	(0.044–0.171)***
Receptive language	0.76	(0.51 –1.01)***	0.379	(0.217–0.576)***	0.105	(0.034–0.194)**
Expressive language	0.68	(0.43–0.94)***	0.354	(0.209–0.539)***	0.117	(0.047–0.204)***
Fine motor	0.40	(0.13–0.68)**	0.318	(0.161–0.492)***	0.086	(0.018–0.177)*
Socio-emotional	0.38	(0.15–0.62)**	0.150	(0.038–0.274)*	0.099	(0.031–0.179)*

*Notes.* ***p < 0.001, **p < 0.01, *p < 0.05. Bootstrapped 95% Confidence Intervals (CIs) adjusted for clustering at the block level (*n* = 500 replications).

For children 19–30 months, the inclusion of parental education in Step 1 and that of the home environment in Step 4 (controlling for all other potential mediators) reduced the effect of SES on cognition and both languages. The reduction in the SES gap for cognition indicated full mediation from parental education and the home environment combined. While the SES gap in fine motor and socio-emotional development for children in this age group was not significant, the quality of the home environment index had a significant effect when entered in the regression (Step 4).

For children 31–42 months, the mediating effects of parental education and the home environment were significant for all scales. However, the SES gap was no longer significant after Step 3 for fine motor development (Supplementary Table 4) and after Step 1 for socio-emotional development (Supplementary Table 4).

## Discussion

Rubio-Codina et al. (2015) previously showed an SES gap in development in a sample of children aged 6–42 months, representative of low- and middle-income families in Bogota. The gap was significant for all scales of the Bayley-III except gross motor, and increased with age. In the current paper, we explored which of a group of variables mediated the effect of SES on cognitive, language, fine motor, and socio-emotional development. Parental education and the quality of the home environment mediated the SES gap in all outcomes examined. Height-for-age reduced the SES gap for receptive and expressive language development only. In contrast, being firstborn did not mediate the effect of SES on any developmental outcome. The important mediation effects of parental education and the quality of the home environment on child development concur with Hamadani et al. (2014).

### Height-For-Age

In rural Bangladesh, Hamadani et al. (2014) showed that pre- and post-natal growth were significant mediators of the effect of poverty on IQ, particularly growth in length before 24 months. In Bogota, height-for-age had a small effect and only mediated the effect of SES on language development. Moreover, unlike in Bangladesh, it did not mediate the effect of maternal education. Differences in poverty and child malnutrition rates between the two samples could account for this inconsistency. The incidence of stunting and low birth weight was much higher in Bangladesh. The Bangladesh study also had multiple measures of growth and longitudinal data, while we only had one set of anthropometric measures.

### Parental Education

Parental education has been shown to be important for child development in other countries in the region, the effect of fathers’ education being usually less important than that of mothers’ (Schady, 2011). Educated mothers are less likely to be depressed, and usually provide a more supportive and better quality home environment, better nutrition for their children, and have higher educational expectations (Green et al., 2009; Hamadani et al., 2014; Walker et al., 2011). In Bogota, maternal education was also more important than parental education in all scales except socio-emotional. The home environment totally mediated the effect of maternal education on cognition and explained the difference in effect of maternal and paternal education. Nonetheless, it only partly mediated the effect of maternal education on language and reduced but did not remove the difference between the effect of fathers’ and mothers’ education. We cannot explain the remaining effect of maternal education with the available data. Father’s education mediated the effect of SES on socio-emotional development and had an independent effect. While we note that more educated fathers are generally more likely to live with the child than less educated fathers in this context, we were unable to explain this relationship; this is an area for further investigation.

### Home Environment

The association of the FCI and child development was particularly large and concurrent with evidence from other countries (Engle et al., 2011). It partially mediated the effect of SES on development and had independent effects, concurring with Hamadani et al. (2014).

The quality of the home environment—defined here as the quantity of play materials and responsive interactions between caregivers and children—is modifiable through interventions (Attanasio et al., 2014; Cooper et al., 2009). Moreover, interventions aimed at improving home stimulation and parenting practices, often delivered through home visits or community groups, have had benefits on children’s development in the short run. Crucially, in those studies with long-term follow-ups, benefits—often larger in more disadvantaged populations—have been sustained into adulthood (Engle et al., 2011). This holds out the promise of these interventions contributing to closing poverty gaps.

The FCI is a relatively new instrument developed from the *Home Observations for Measurement of the Environment* (HOME) for use at large scale. It has shown good predictive validity of child development in earlier studies (Hamadani et al., 2010). Our findings further support its use as a survey-based indicator of the quality of the home environment.

In percentage units, the largest reduction in the SES gap was in language: 88% for receptive and 69% for expressive language. Language has consistently been found to be affected by SES in Latin America (Schady et al., 2015) and in other areas of the world (Fernald et al., 2011 for Madagascar, for example).

The effects of maternal education tended to increase with age, whereas the effect of the home environment became significant at 19–30 months, and then plateaued. This could reflect greater difficulty in assessing children at young ages, although internal consistency remained stable by age. It could also reflect the cumulative influence of these variables over time on child development, and is an area for further research. However, these differences in the mediation effect for cognition and language as a percentage of the (age-increasing) size of the SES gap between children in the middle and the older age groups were not statistically significant.

Limitations of the study included cross-sectional and observational (rather than experimental) data. This implied that causal inferences on mediation cannot be drawn. Furthermore, it is not possible to know whether earlier exposures have later effects. In Bangladesh, for example, growth in the first 2 years and stimulation at 18 months had long-term effects on IQ at 5 years (Hamadani et al., 2014). Moreover, variables in the analysis may be simultaneously influenced by other variables (parental intellectual or socio-emotional ability, for example) not directly controlled for. Another limitation was the lack of reliability data for the FCI, although the associations reported suggested poor measurement was not an issue. Finally, the Bayley-III was not standardized for Colombia. However, the translated version showed good internal and test–retest reliability and was related to other variables in theoretically expected ways. Moreover, we used internally standardized scores rather than the scores derived from the normative sample. In a contemporaneous study in Colombia (not published) the test also showed acceptable levels of predictive validity with school readiness and vocabulary at 5 years of age, further suggesting validity of the Bayley-III in this population.

Study strengths were the large sample and high-quality measures of development by domain across a 3-year age range. Findings can inform the design of interventions that promote early childhood development and stimulate more research in interventions aimed at improving the quality of the home environment. Our results suggested that there may be high returns to intervening early, and targeting the quality of the home environment with particular focus on language and cognition—the areas most affected by poverty.

In conclusion, the findings showed that large developmental deficits associated with poverty from early ages were strongly mediated by parental education, especially maternal education, and the quality of the home environment. Height-for-age mediated a small amount of the deficit in language only. Interventions aimed at the quality of the home environment should be effective at reducing developmental deficits associated with poverty.

## Supplementary Material

Supplementary material

## Note

1. Percentage reductions were computed by dividing the change in the SES gap after controlling for parental education (reported in Table 4, panel I) over the size of the gap prior to the introduction of these variables (reported in Table 3).

**Supplemental material:** The online tables are available at http://jbd.sagepub.com/supplemental.

## References

[bibr1-0165025415626515] Attanasio O. P. Fernández C. Fitzsimons E. O. Meghir C. Grantham-McGregor S. M Rubio-Codina M. (2014). Using the infrastructure of a conditional cash transfer program to deliver a scalable integrated early child development program in Colombia: cluster randomized controlled trail. British Medical Journal, 349, g5785. 25266222 10.1136/bmj.g5785 PMC4179481

[bibr2-0165025415626515] Bayley N. (2006). Bayley Scales of Infant and Toddler Development – Third Edition. San Antonio, TX: Harcourt Assessment.

[bibr3-0165025415626515] Cooper P. J. Tomlinson M. Swartz L. Landman M. Molteno C. Stein A Murray L. (2009). Improving quality of mother-infant relationship and infant attachment in socioeconomically deprived community in South Africa: Randomized controlled trail. British Medical Journal, 338, b974. 19366752 10.1136/bmj.b974 PMC2669116

[bibr4-0165025415626515] Dearing E. Hamilton L. C. (2006). Contemporary advances and classic advice for analyzing mediating and moderating variables. Monographs of the Society for Research in Child Development, 71(3), 88–104.

[bibr5-0165025415626515] Efron B. (1982). The Jackknife, the Bootstrap, and Other Resampling Plans. Society of Industrial and Applied Mathematics CBMS-NSF Monographs, 38.

[bibr6-0165025415626515] Engle P. L. Fernald L. C. Alderman H. Behrman J. O’Gara C. Yousafzai A., … the Global Child Development Steering Group. (2011). Strategies for reducing inequalities and improving developmental outcomes for young children in low-income and middle-income countries. The Lancet, 378(9799), 1339–1353. 10.1016/S0140-6736(11)60889-1 21944378

[bibr7-0165025415626515] Fernald L. C. Kariger P. Hidrobo M. Gertler P. J. (2012). Socioeconomic gradients in child development in very young children: Evidence from India, Indonesia, Peru, and Senegal. Proceedings of the National Academy of Sciences, 109(2), 17273–17280. 10.1073/pnas.1121241109 PMC3477389 23045688

[bibr8-0165025415626515] Fernald L. Weber A. Galasso E. Ratsifandrihamanana L. (2011). Socioeconomic gradients and child development in a very low income population: Evidence from Madagascar. Developmental Science, 14(4), 832–847. 21676102 10.1111/j.1467-7687.2010.01032.x

[bibr9-0165025415626515] Frongillo E. A. Sywulka S. M Kariger P. (2003). UNICEF psychosocial care indicators project. Final report to UNICEF, mimeo Cornell University.

[bibr10-0165025415626515] Green C.M. Berkule S.B. Dreyer B. P. Fierman A. H. Huberman H. S. Klass P. E … Mendelsohn A. L. (2009). Maternal literacy and associations between education and the cognitive home environment in low-income families. Archives of Pediatrics & Adolescent Medicine, 163(9), 832–847. 19736337 10.1001/archpediatrics.2009.136 PMC3083977

[bibr11-0165025415626515] Hackman D. A. Farah M. J. Meaney J. (2010). Socioeconomic status and the brain: mechanistic insights from human and animal research. Nature Reviews Neuroscience, 11, 651–659. 20725096 10.1038/nrn2897 PMC2950073

[bibr12-0165025415626515] Hamadani J. D. Tofail F. Hilaly A. Huda S. N. Engle P. Grantham-McGregor S. M. (2010). Use of the family care indicators and their relationship with child development in Bangladesh. Journal of Health Population and Nutrition, 28(1), 23–33. 10.3329/jhpn.v28i1.4520 PMC2975843 20214083

[bibr13-0165025415626515] Hamadani J. D. Tofail F. Huda S. N. Alam D. S. Ridout D. A. Attanasio O. Grantham-McGregor S. M. (2014). Cognitive deficit and poverty in the first 5 years of childhood in Bangladesh, Pediatrics, 134(4), e1001–e1008. 25266433 10.1542/peds.2014-0694

[bibr14-0165025415626515] MacKinnon D. P. Lockwood C. M. Hoffman J. M. West S. G. Sheets V. (2002). A comparison of methods to test mediation and other intervening variable effects. Psychological Methods, 7(1), 83–104. 11928892 10.1037/1082-989x.7.1.83 PMC2819363

[bibr15-0165025415626515] MacKinnon D. P. Warsi G. Dwyer J. H. (1995). A simulation study of mediated effect measures. Multivariate Behavioral Research, 30(1), 41–62. 20157641 10.1207/s15327906mbr3001_3 PMC2821114

[bibr16-0165025415626515] Martorell R. Horta B. L Adair L. S. Stein A. D. Richter L. Fall C. H., … the Consortium on Health Orientated Research in Transitional Societies Group. (2010). Weight gain in the first two years of life is an important predictor of schooling outcomes in pooled analyses from five birth cohorts form low- and middle-income countries. Journal of Nutrition, 140(2), 348–354. 10.3945/jn.109.112300 PMC2806888 20007336

[bibr17-0165025415626515] Naudeau S. Martinez S. Premand P. Filmer D. (2011). Cognitive development among young children in low-income countries In Alderman H. (Ed.), No Small Matter. The impact of Poverty, Shocks, and Human Capital Investments in Early Childhood Development (pp. 9–50). Washington D.C., US: The World Bank.

[bibr18-0165025415626515] Rubio-Codina M. Attanasio O. Meghir C. Varela N. Grantham-McGregor S. (2015). The socioeconomic gradient of child development: Cross-sectional evidence from children 6-42 months in Bogota. Journal of Human Resources, 50(2), 465–483.

[bibr19-0165025415626515] Schady N. (2011). Parents’ education, mothers’ vocabulary, and cognitive development in early childhood: longitudinal evidence from Ecuador. American Journal of Public Health, 101(12), 2299–2307. 22021308 10.2105/AJPH.2011.300253 PMC3222428

[bibr20-0165025415626515] Schady N. Behrman J. Araujo M. C. Azuero R. Bernal R. Bravo D. … Vakis R. (2015). Wealth gradients in early childhood cognitive development in five Latin American countries. Journal of Human Resources, 50(2), 447–464. 10.3368/jhr.50.2.446 PMC4431591 25983344

[bibr21-0165025415626515] Walker S. P. Wachs T. D. Grantham-McGregor S. Black M. M. Huffman S. L. Baker-Henningham H. … Richter L. (2011). Inequality in early childhood: risk and protective factors for early child development. The Lancet, 378(9799), 1325–1338. 10.1016/S0140-6736(11)60555-2 21944375

[bibr22-0165025415626515] WHO. (1983). Measuring change in nutritional status: Guidelines for assessing the nutritional impact of supplementary feeding programmes for vulnerable groups. Geneva: WHO.

