# Supplementary material for: Mediating pathways in the socio-economic gradient of child development: Evidence from children 6–42 months in Bogota
Source: Int J Behav Dev. 2016 Jun 17;40(6):483–91. doi: 10.1177/0165025415626515 (PMC5102093; doi:10.1177/0165025415626515)
Supplement: Supplementary material [file MSJ626515_supplementary_material.pdf]

**Supplementary Table 1. Correlations across predictor, developmental outcomes, and potential mediators by age.**

| | Cognition | Receptive language | Expressive language | Fine motor | Socio-emotional | SES (wealth) index |
|---|---|---|---|---|---|---|
| **I. Children 6-18 months** (N =451) | | | | | | |
| **Predictor** | | | | | | |
| SES (wealth) index | 0.110* | 0.141** | 0.206*** | 0.092 | 0.091 | 1 |
| **Mediators** | | | | | | |
| Mother's education | 0.061 | 0.172** | 0.159** | 0.058 | 0.053 | 0.460*** |
| Father's education | 0.037 | 0.063 | 0.125** | -0.018 | 0.091* | 0.283*** |
| Firstborn | 0.030 | 0.137 | 0.098 | 0.056 | 0.054 | 0.069 |
| Height-for-age | -0.047 | 0.048 | 0.041 | 0.025 | -0.050 | 0.02 |
| Home environment index | 0.136** | 0.198*** | 0.125** | 0.076 | 0.047 | 0.398*** |
| **II. Children 19-30 months** (N =460) | | | | | | |
| **Predictor** | | | | | | |
| SES (wealth) index | 0.226*** | 0.158*** | 0.189*** | 0.086 | 0.079 | 1 |
| **Mediators** | | | | | | |
| Mother's education | 0.247*** | 0.189*** | 0.214*** | 0.096* | 0.04 | 0.467*** |
| Father's education | 0.179*** | 0.128*** | 0.139** | 0.061 | 0.094* | 0.338*** |
| Firstborn | 0.071 | 0.100* | 0.126** | -0.058 | -0.012 | 0.160*** |
| Height-for-age | 0.065 | 0.074 | 0.046 | 0.029 | 0.006 | 0.169*** |
| Home environment index | 0.273*** | 0.267*** | 0.254*** | 0.139** | 0.201*** | 0.462*** |
| **III. Children 31-42 months** (N =419) | | | | | | |
| **Predictor** | | | | | | |
| SES (wealth) index | 0.354*** | 0.303*** | 0.267*** | 0.189*** | 0.178*** | 1 |
| **Mediators** | | | | | | |
| Mother's education | 0.362*** | 0.374*** | 0.328*** | 0.262*** | 0.155** | 0.499*** |
| Father's education | 0.209*** | 0.145** | 0.155** | 0.102* | 0.158** | 0.332*** |
| Firstborn | 0.081 | 0.062 | 0.114* | -0.005 | 0.056 | 0.125* |
| Height-for-age | 0.164** | 0.118* | 0.160** | 0.143** | 0.014 | 0.119* |
| Home environment index | 0.371*** | 0.355*** | 0.343*** | 0.223*** | 0.276*** | 0.457*** |

Notes. All variables are Z-scores, except for firstborn (indicator). ***p<0.001, **p<0.01, *p<0.05

**Supplementary Table 2: Regression coefficients and 95% confidence intervals for the effect of the SES gap and potential mediators on Bayley-III Z-scores. Children 6-18 months (N =451).**

| | Step 0 | Step 1 | Step 2 | Step 3 | Step 4 |
|---|---|---|---|---|---|
| | τ (95% CI) | τ', β (95% CI) | τ', β (95% CI) | τ', β (95% CI) | τ', β (95% CI) |
| **I. Cognition** | | | | | |
| Step 0: SES gap | 0.26 (-0.01-0.53) | 0.25 (-0.03-0.52) | 0.24 (-0.03-0.52) | 0.25 (-0.03-0.53) | 0.20 (-0.09-0.49) |
| Step 1: Mother's education | | -0.00 (-0.11-0.11) | -0.02 (-0.12-0.09) | -0.01 (-0.12-0.09) | -0.04 (-0.15-0.07) |
| Father's education | | 0.01 (-0.07-0.10) | 0.02 (-0.06-0.11) | 0.02 (-0.06-0.11) | 0.02 (-0.06-0.11) |
| Step 2: Firstborn | | | 0.11 (-0.07-0.29) | 0.11 (-0.06-0.29) | 0.09 (-0.08-0.27) |
| Step 3: Height-for-age | | | | -0.02 (-0.11-0.07) | -0.02 (-0.11-0.07) |
| Step 4: Home environment index | | | | | 0.11 (-0.02-0.24) |
| **II. Receptive language** | | | | | |
| Step 0: SES gap | 0.20 (-0.04-0.45) | 0.03 (-0.24-0.29) | 0.03 (-0.23-0.29) | 0.01 (-0.25-0.27) | -0.05 (-0.30-0.20) |
| Step 1: Mother's education | | 0.15 (0.05-0.25)** | 0.11 (0.01-0.21)* | 0.11 (0.01-0.21)* | 0.08 (-0.03-0.18) |
| Father's education | | 0.01 (-0.08-0.11) | 0.03 (-0.06-0.13) | 0.04 (-0.05-0.13) | 0.03 (-0.06-0.13) |
| Step 2: Firstborn | | | 0.29 (0.12-0.46)** | 0.27 (0.10-0.44)** | 0.25 (0.08-0.42)** |
| Step 3: Height-for-age | | | | 0.08 (0.00-0.17)* | 0.08 (-0.00-0.16) |
| Step 4: Home environment index | | | | | 0.13 (0.02-0.24)* |
| **III. Expressive language** | | | | | |
| Step 0: SES gap | 0.43 (0.16-0.70)** | 0.28 (-0.03-0.59) | 0.28 (-0.02-0.58) | 0.27 (-0.03-0.58) | 0.26 (-0.05-0.57) |
| Step 1: Mother's education | | 0.09 (-0.02-0.21) | 0.07 (-0.05-0.18) | 0.06 (-0.05-0.18) | 0.06 (-0.06-0.17) |
| Father's education | | 0.06 (-0.05-0.17) | 0.08 (-0.03-0.19) | 0.08 (-0.03-0.19) | 0.08 (-0.03-0.19) |
| Step 2: Firstborn | | | 0.23 (0.04-0.41)* | 0.21 (0.03-0.39)* | 0.21 (0.03-0.39)* |
| Step 3: Height-for-age | | | | 0.05 (-0.02-0.11) | 0.05 (-0.02-0.11) |
| Step 4: Home environment index | | | | | 0.03 (-0.10-0.15) |
| **IV. Fine motor** | | | | | |
| Step 0: SES gap | 0.25 (0.01-0.49)* | 0.25 (-0.01-0.51) | 0.25 (-0.01-0.51) | 0.25 (-0.02-0.51) | 0.21 (-0.05-0.46) |
| Step 1: Mother's education | | 0.03 (-0.10-0.15) | 0.01 (-0.11-0.14) | 0.01 (-0.11-0.14) | -0.01 (-0.13-0.12) |
| Father's education | | -0.04 (-0.14-0.07) | -0.03 (-0.13-0.07) | -0.03 (-0.13-0.07) | -0.03 (-0.14-0.07) |
| Step 2: Firstborn | | | 0.10 (-0.08-0.28) | 0.10 (-0.09-0.28) | 0.08 (-0.10-0.27) |
| Step 3: Height-for-age | | | | 0.01 (-0.09-0.11) | 0.01 (-0.09-0.11) |
| Step 4: Home environment index | | | | | 0.08 (-0.04-0.20) |
| **V. Socio-emotional** | | | | | |
| Step 0: SES gap | 0.30 (0.03-0.57)* | 0.24 (-0.06-0.55) | 0.24 (-0.07-0.56) | 0.25 (-0.06-0.56) | 0.26 (-0.06-0.58) |
| Step 1: Mother's education | | 0.01 (-0.11-0.12) | -0.01 (-0.13-0.11) | -0.01 (-0.13-0.11) | -0.01 (-0.13-0.12) |
| Father's education | | 0.07 (-0.02-0.16) | 0.08 (-0.02-0.17) | 0.08 (-0.02-0.17) | 0.08 (-0.02-0.17) |
| Step 2: Firstborn | | | 0.14 (-0.04-0.32) | 0.16 (-0.03-0.34) | 0.16 (-0.03-0.34) |
| Step 3: Height-for-age | | | | -0.06 (-0.13-0.02) | -0.06 (-0.13-0.02) |
| Step 4: Home environment index | | | | | -0.01 (-0.13-0.10) |

Notes. ***p<0.001, **p<0.01, *p<0.05. For each outcome (panel), columns represent separate regressions. All variables are Z-scores, except for firstborn (indicator). All regressions control for wealth quartile dummies, child's age and sex, and tester effects. 95% Confidence Intervals (CI) are adjusted for clustering at the block level.

**Supplementary Table 3: Regression coefficients and 95% confidence intervals for the effect of the SES gap and potential mediators on Bayley-III Z-scores. Children 19-30 months (N =460).**

| | Step 0 | Step 1 | Step 2 | Step 3 | Step 4 |
|---|---|---|---|---|---|
| | τ (95% CI) | τ', β (95% CI) | τ', β (95% CI) | τ', β (95% CI) | τ', β (95% CI) |
| **I. Cognition** | | | | | |
| Step 0: SES gap | 0.55 (0.32-0.78)*** | 0.30 (0.01-0.58)* | 0.29 (0.00-0.57)* | 0.27 (-0.03-0.56) | 0.12 (-0.19-0.43) |
| Step 1: Mother's education | | 0.12 (-0.00-0.25)* | 0.11 (-0.02-0.24) | 0.11 (-0.02-0.24) | 0.06 (-0.08-0.19) |
| Father's education | | 0.12 (0.01-0.23)* | 0.12 (0.01-0.23)* | 0.12 (0.01-0.23)* | 0.10 (-0.01-0.21) |
| Step 2: Firstborn | | | 0.08 (-0.09-0.25) | 0.07 (-0.10-0.24) | 0.07 (-0.10-0.23) |
| Step 3: Height-for-age | | | | 0.05 (-0.05-0.14) | 0.04 (-0.05-0.14) |
| Step 4: Home environment index | | | | | 0.19 (0.09-0.28)*** |
| **II. Receptive language** | | | | | |
| Step 0: SES gap | 0.30 (0.06-0.55)* | 0.11 (-0.19-0.41) | 0.09 (-0.20-0.39) | 0.07 (-0.22-0.37) | -0.10 (-0.40-0.21) |
| Step 1: Mother's education | | 0.10 (-0.01-0.21) | 0.08 (-0.04-0.20) | 0.08 (-0.04-0.20) | 0.01 (-0.11-0.13) |
| Father's education | | 0.08 (-0.03-0.19) | 0.09 (-0.02-0.20) | 0.09 (-0.02-0.20) | 0.07 (-0.04-0.18) |
| Step 2: Firstborn | | | 0.18 (0.01-0.35)* | 0.17 (-0.00-0.34) | 0.16 (-0.01-0.33) |
| Step 3: Height-for-age | | | | 0.04 (-0.05-0.12) | 0.03 (-0.05-0.12) |
| Step 4: Home environment index | | | | | 0.22 (0.13-0.31)*** |
| **III. Expressive language** | | | | | |
| Step 0: SES gap | 0.41 (0.17-0.65)*** | 0.21 (-0.08-0.50) | 0.19 (-0.09-0.48) | 0.19 (-0.11-0.48) | 0.04 (-0.27-0.35) |
| Step 1: Mother's education | | 0.11 (0.01-0.21)* | 0.08 (-0.02-0.19) | 0.08 (-0.02-0.19) | 0.02 (-0.08-0.13) |
| Father's education | | 0.08 (-0.02-0.17) | 0.08 (-0.01-0.18) | 0.08 (-0.01-0.18) | 0.07 (-0.03-0.16) |
| Step 2: Firstborn | | -0.07 (-0.45-0.31) | -0.09 (-0.46-0.28) | -0.09 (-0.46-0.28) | -0.03 (-0.40-0.34) |
| Step 3: Height-for-age | | | 0.21 (0.03-0.38)* | 0.20 (0.03-0.38)* | 0.20 (0.03-0.37)* |
| Step 4: Home environment index | | | | 0.01 (-0.08-0.10) | 0.01 (-0.08-0.10) |
| **IV. Fine motor** | | | | | |
| Step 0: SES gap | 0.13 (-0.09-0.36) | 0.08 (-0.21-0.36) | 0.09 (-0.19-0.37) | 0.07 (-0.22-0.35) | -0.03 (-0.34-0.29) |
| Step 1: Mother's education | | 0.01 (-0.10-0.13) | 0.03 (-0.10-0.15) | 0.03 (-0.10-0.15) | -0.01 (-0.12-0.11) |
| Father's education | | 0.04 (-0.05-0.13) | 0.04 (-0.05-0.13) | 0.04 (-0.05-0.13) | 0.03 (-0.06-0.12) |
| Step 2: Firstborn | | | -0.11 (-0.30-0.09) | -0.12 (-0.31-0.08) | -0.12 (-0.31-0.07) |
| Step 3: Height-for-age | | | | 0.04 (-0.04-0.12) | 0.04 (-0.04-0.12) |
| Step 4: Home environment index | | | | | 0.12 (0.01-0.23)* |
| **V. Socio-emotional** | | | | | |
| Step 0: SES gap | 0.20 (-0.04-0.45) | 0.14 (-0.12-0.39) | 0.14 (-0.11-0.40) | 0.13 (-0.12-0.39) | -0.03 (-0.29-0.22) |
| Step 1: Mother's education | | 0.01 (-0.10-0.12) | 0.02 (-0.10-0.13) | 0.02 (-0.10-0.13) | -0.05 (-0.16-0.06) |
| Father's education | | 0.06 (-0.03-0.16) | 0.06 (-0.03-0.15) | 0.06 (-0.03-0.15) | 0.04 (-0.05-0.13) |
| Step 2: Firstborn | | | -0.04 (-0.23-0.14) | -0.05 (-0.24-0.14) | -0.05 (-0.24-0.13) |
| Step 3: Height-for-age | | | | 0.02 (-0.08-0.11) | 0.01 (-0.08-0.11) |
| Step 4: Home environment index | | | | | 0.21 (0.11-0.31)*** |

Notes. ***p<0.001, **p<0.01, *p<0.05. For each outcome (panel), columns represent separate regressions. All variables are Z-scores, except for firstborn (indicator). All regressions control for wealth quartile dummies, child's age and sex, and tester effects. 95% Confidence Intervals (CI) are adjusted for clustering at the block level.

## Supplementary Table 4: Regression coefficients and 95% confidence intervals for the effect of the SES gap and potential mediators on Bayley-III Z-scores. Children 31-42 months (N =419).

| | Step 0 | Step 1 | Step 2 | Step 3 | Step 4 |
|---|---|---|---|---|---|
| | τ (95% CI) | τ', β (95% CI) | τ', β (95% CI) | τ', β (95% CI) | τ', β (95% CI) |
| **I. Cognition** | | | | | |
| Step 0: SES gap | 0.81 (0.55-1.08)*** | 0.45 (0.16-0.73)** | 0.45 (0.16-0.73)** | 0.42 (0.14-0.70)** | 0.32 (0.04-0.60)* |
| Step 1: Mother's education | | 0.24 (0.13-0.35)*** | 0.24 (0.13-0.35)*** | 0.24 (0.13-0.35)*** | 0.19 (0.09-0.30)** |
| Father's education | | 0.06 (-0.03-0.16) | 0.06 (-0.03-0.16) | 0.06 (-0.03-0.16) | 0.05 (-0.05-0.14) |
| Step 2: Firstborn | | | 0.00 (-0.16-0.17) | -0.01 (-0.16-0.15) | -0.02 (-0.18-0.14) |
| Step 3: Height-for-age | | | | 0.12 (0.03-0.21)** | 0.12 (0.04-0.21)** |
| Step 4: Home environment index | | | | | 0.16 (0.07-0.24)*** |
| **II. Receptive language** | | | | | |
| Step 0: SES gap | 0.76 (0.51-1.01)*** | 0.38 (0.11-0.65)** | 0.39 (0.11-0.66)** | 0.37 (0.10-0.64)** | 0.27 (-0.00-0.54) |
| Step 1: Mother's education | | 0.30 (0.19-0.41)*** | 0.31 (0.19-0.43)*** | 0.31 (0.19-0.42)*** | 0.26 (0.14-0.38)*** |
| Father's education | | -0.01 (-0.12-0.11) | -0.01 (-0.13-0.10) | -0.02 (-0.13-0.10) | -0.03 (-0.15-0.09) |
| Step 2: Firstborn | | | -0.08 (-0.26-0.10) | -0.09 (-0.27-0.09) | -0.11 (-0.29-0.07) |
| Step 3: Height-for-age | | | | 0.07 (-0.02-0.15) | 0.07 (-0.01-0.14) |
| Step 4: Home environment index | | | | | 0.16 (0.05-0.27)** |
| **III. Expressive language** | | | | | |
| Step 0: SES gap | 0.68 (0.43-0.94)*** | 0.33 (0.03-0.62)* | 0.33 (0.03-0.62)* | 0.30 (0.01-0.59)* | 0.18 (-0.11-0.48) |
| Step 1: Mother's education | | 0.26 (0.16-0.37)*** | 0.26 (0.15-0.37)*** | 0.26 (0.15-0.36)*** | 0.20 (0.09-0.31)*** |
| Father's education | | 0.02 (-0.09-0.13) | 0.02 (-0.09-0.13) | 0.02 (-0.10-0.13) | 0.00 (-0.11-0.12) |
| Step 2: Firstborn | | -0.23 (-0.63-0.16) | -0.23 (-0.63-0.16) | -0.21 (-0.61-0.19) | -0.22 (-0.62-0.17) |
| Step 3: Height-for-age | | | 0.03 (-0.15-0.22) | 0.02 (-0.15-0.20) | 0.01 (-0.17-0.19) |
| Step 4: Home environment index | | | | 0.11 (0.04-0.19)** | 0.11 (0.04-0.18)** |
| **IV. Fine motor** | | | | | |
| Step 0: SES gap | 0.40 (0.13-0.68)** | 0.08 (-0.23-0.40) | 0.10 (-0.22-0.42) | 0.07 (-0.25-0.39) | -0.01 (-0.33-0.30) |
| Step 1: Mother's education | | 0.24 (0.13-0.36)*** | 0.26 (0.15-0.38)*** | 0.26 (0.14-0.37)*** | 0.22 (0.10-0.34)*** |
| Father's education | | 0.00 (-0.10-0.11) | -0.01 (-0.11-0.09) | -0.01 (-0.11-0.09) | -0.02 (-0.13-0.08) |
| Step 2: Firstborn | | | -0.19 (-0.38--0.00) | -0.20 (-0.39--0.01) | -0.22 (-0.41--0.02) |
| Step 3: Height-for-age | | | | 0.12 (0.02-0.22)* | 0.12 (0.03-0.22)* |
| Step 4: Home environment index | | | | | 0.13 (0.02-0.25)* |
| **V. Socio-emotional** | | | | | |
| Step 0: SES gap | 0.38 (0.15-0.62)* | 0.23 (-0.03-0.50) | 0.23 (-0.04-0.49) | 0.23 (-0.04-0.50) | 0.13 (-0.16-0.42) |
| Step 1: Mother's education | | 0.06 (-0.03-0.15) | 0.05 (-0.04-0.15) | 0.05 (-0.04-0.15) | 0.01 (-0.08-0.11) |
| Father's education | | 0.10 (0.01-0.19)* | 0.10 (0.01-0.19)* | 0.10 (0.01-0.19)* | 0.09 (-0.01-0.18) |
| Step 2: Firstborn | | | 0.05 (-0.14-0.24) | 0.05 (-0.15-0.25) | 0.04 (-0.16-0.24) |
| Step 3: Height-for-age | | | | -0.02 (-0.11-0.07) | -0.02 (-0.11-0.06) |
| Step 4: Home environment index | | | | | 0.15 (0.05-0.25)** |

Notes. ***p<0.001, **p<0.01, *p<0.05. For each outcome (panel), columns represent separate regressions. All variables are Z-scores, except for firstborn (indicator). All regressions control for wealth quartile dummies, child's age and sex, and tester effects. 95% Confidence Intervals (CI) are adjusted for clustering at the block level.
